# Criteria Used to Determine Platinum Eligibility in Patients with Metastatic Urothelial Carcinoma: Results of a Physician Survey in Five European Countries

**DOI:** 10.1016/j.euros.2025.08.009

**Published:** 2025-09-23

**Authors:** Shilpa Gupta, Thomas Powles, Mairead Kearney, Laura Panattoni, Natalie Land, Thomas Flottemesch, Patrick Sullivan, Melissa Kirker, Murtuza Bharmal, Silke Guenther, Nuno Costa, Enrique Grande

**Affiliations:** a Taussig Cancer Institute, Cleveland Clinic Foundation, Cleveland, OH, USA; b Barts Cancer Institute, Experimental Cancer Medicine Centre, Queen Mary University of London, St Bartholomew’s Hospital, London, UK; c Merck Healthcare KGaA, Darmstadt, Germany; d PRECISIONheor, New York, NY, USA; e Pfizer, New York, NY, USA; f EMD Serono, Research & Development Institute, Inc., Billerica, MA, USA, an affiliate of Merck KGaA; g Pfizer, Porto Salvo, Portugal; h Medical Oncology Department, MD Anderson Cancer Center Madrid, Madrid, Spain

**Keywords:** Bladder cancer, Platinum-based chemotherapy, Treatment decision-making, Urothelial carcinoma, Physician survey, EU5, Platinum eligibility

## Abstract

**Background and objective:**

Although, for decades, first-line treatment for metastatic urothelial carcinoma (mUC) has been based on eligibility for platinum-based chemotherapy (cisplatin or carboplatin), the platinum-ineligibility criteria used by European physicians in real-world practice are unclear. This study assessed the factors and clinical thresholds used by European physicians to determine platinum ineligibility and alignment with the published US thresholds.

**Methods:**

Physicians in France, Germany, Italy, Spain, and the UK completed a quantitative online survey from August to September 2022. Physicians reported percentages of patients perceived as platinum eligible and factors considered when determining platinum ineligibility. A previous US-based survey was adapted to identify European consensus thresholds. Differences between European and US consensus thresholds were assessed using *t* tests.

**Key findings and limitations:**

In total, 503 physicians (69% oncologists and 31% urologists) completed the survey. Respondents reported that 77% of treated patients with mUC were platinum eligible. European (vs US) consensus thresholds for platinum ineligibility were creatinine clearance of <25 ml/min (vs <30 ml/min) or peripheral neuropathy grade ≥3 (vs grade ≥2). Consensus thresholds were the same for Eastern Cooperative Oncology Group performance status ≥3 and New York Heart Association class III. The European consensus age threshold was 80 yr (vs no threshold in the US study). Limitations include a potential bias due to voluntary participation.

**Conclusions and clinical implications:**

European physicians considered most patients with mUC to be platinum eligible. Ineligibility criteria were broadly consistent with US results, except for age. Standardization and wider use of these criteria could support decision-making, reduce variations in care, and improve patient outcomes.

**Patient summary:**

A survey of doctors from five European countries (France, Germany, Italy, Spain, and the UK) found that most people with metastatic urothelial cancer (77%) should be able to receive platinum-based chemotherapy. The characteristics considered by European doctors to decide whether a person can receive platinum-based chemotherapy were similar to those reported previously by US doctors, except for age. Specifically, most European doctors (67%) reported that they may be reluctant to prescribe platinum-based chemotherapy to a >80-yr-old person with metastatic urothelial cancer, whereas US doctors reported that they did not consider age in their decision.

## Take Home Message

European physicians reported that 77% of patients with metastatic urothelial carcinoma were platinum eligible. The ineligibility criteria were broadly consistent with US-based studies, except for age. Standardization and wider use of these criteria could support decision-making and improve patient outcomes.

## 1 Introduction

Bladder cancer is the tenth most common malignancy worldwide, accounting for approximately 573 000 new cases and 213 000 deaths in 2020 [1,2]. Among men, bladder cancer is the fourth most common cancer in the USA, France, Germany, Italy, and Spain, and the seventh most common in the UK [3,4]. Locally advanced and metastatic urothelial carcinoma (mUC) are considered incurable and have a 5-yr survival rate of 8% [5].

European and US clinical guidelines for first-line (1L) treatment classify patients with mUC into three groups: eligible for cisplatin-based chemotherapy, ineligible for cisplatin-based chemotherapy but eligible for carboplatin-based chemotherapy, and ineligible for any platinum-based chemotherapy [[6], [7], [8]]. Following platinum-based chemotherapy, maintenance treatment with avelumab, an anti–PD-L1 immune checkpoint inhibitor, is the standard 1L treatment for patients with mUC without disease progression, based on level 1 evidence [[6], [7], [8], [9]]. In Europe, atezolizumab and pembrolizumab are also recommended options for 1L treatment of cisplatin-ineligible patients with PD-L1+ tumors [6,7]. In the USA, approval of 1L pembrolizumab is restricted to platinum-ineligible patients, regardless of PD-L1 expression, whereas the indication for atezolizumab was voluntarily withdrawn [10,11].

Despite guideline consensus in the USA and Europe, growing evidence shows substantial underuse of 1L systemic treatment for mUC and variations in the use of 1L platinum-based chemotherapy [[12], [13], [14], [15], [16], [17]]. A recent review concluded that many patients with mUC who are eligible for 1L systemic treatment may not be receiving guideline-concordant care [12,18], negatively impacting clinical outcomes. Underuse of guideline-recommended therapy may be partly driven by physicians’ uncertainty about platinum eligibility. In 2021, European Association of Urology (EAU) guidelines defined platinum ineligibility in patients with mUC as performance status (PS) >2, a glomerular filtration rate of <30 ml/min, a combination of PS 2 and glomerular filtration rate of <60 ml/min, or a comorbidity of grade >2 [7]. However, the criteria used by European physicians to define platinum ineligibility in real-world practice are unclear. The aim of this study was to identify the criteria used by physicians in five European countries to define platinum ineligibility and their alignment with previously reported US-based results [19,20].

Recently, results from the pivotal EV-302 trial demonstrated that enfortumab vedotin plus pembrolizumab outperformed platinum-based chemotherapy in the 1L treatment of patients with mUC. However, it must be noted that EV-302 only enrolled patients who were “platinum eligible,” and hence it is important to understand the decision-making patterns of physicians who will adopt this new standard of care [21].

## 2 Patients and methods

### 2.1 Setting and population

A self-administered web-based survey was completed by practicing oncologists and urologists from France, Germany, Italy, Spain, and the UK who treated patients with mUC. Participants were recruited via third-party research databases (Sago, Iselin, NJ, USA). Country-specific quotas were set to recruit a proportional mix of provider types, with a target of 100 participants per country. Oncologists and urologists were eligible to participate if they had a medical degree, specialized in oncology or urology, had practiced medicine for ≥3 yr, spent ≥50% of their week providing direct patient care, and treated two or more patients with mUC per month. Recruitment was double blinded between participants and the study sponsor.

### 2.2 Data collection

The survey was translated and pilot tested with 20 physicians (*n* = 4 per country). Subsequently, potential participants received a link to the survey website with a study overview and a detailed information statement. Those who agreed to participate completed a series of questions to determine whether they met the inclusion criteria. Qualified participants who completed the full survey received monetary compensation. Recruitment and primary survey data collection were completed between August and September 2022. An independent institutional review board organization (Advarra) reviewed all study materials and granted exemption (protocol number 00046328). All participants provided written informed consent.

Physician demographic and clinical practice characteristics, including age, sex, years in clinical practice, practice setting, and average number of patients with mUC treated per month were collected. Physicians were asked to report the percentage of their patients with mUC who had received 1L systemic anticancer treatment in the past 6 mo and the proportions they perceived as cisplatin eligible, cisplatin ineligible but carboplatin eligible, and platinum ineligible (ie, ineligible for cisplatin and carboplatin; Supplementary Table 1). To understand the factors considered when deciding to administer platinum-based chemotherapy, physicians were asked to allocate a total of 100 points (0, lowest importance; 100, highest importance) across 11 clinical factors (advanced age, hearing loss, impaired cardiac function, impaired pulmonary function, patient refusal, peripheral neuropathy, poor PS, poor renal function, prior immunotherapy, prior platinum therapy, and other comorbid conditions) chosen based on a prior survey [14].

To elicit clinical thresholds used to define platinum ineligibility, six questions from two previous surveys of US physicians were included [19,20]. Physicians selected thresholds for age, creatinine clearance (CrCl), Eastern Cooperative Oncology Group (ECOG) PS, New York Heart Association (NYHA) heart failure class, and peripheral neuropathy grade. Questions were of multiple choice type and included a range of threshold options, including “none” and “other.”

### 2.3 Statistical analysis

Demographic and clinical practice characteristics were reported for the full sample and by country. Differences across countries were analyzed using Kruskal-Wallis rank sum test for continuous data and Fisher’s exact test for count data. Data were visualized as box and whisker plots for percentage of patients whom physicians reported as cisplatin eligible, carboplatin eligible only, and platinum ineligible for the overall sample and by country. Additionally, box and whisker plots were used to represent the points allocated to the 11 decision factors. Full sample results for the six questions that elicited the thresholds for platinum ineligibility were compared with prior US results [19,20]; differences between the full sample and previous US results for each criterion were assessed using Fisher’s exact test. Additionally, Fisher’s exact test was used to assess differences between European countries for each criterion. We reported the cumulative percentage of physicians who would not treat a patient at, above, or below each threshold (depending on the question) compared with thresholds identified in the 2022 US survey [20]. Consensus thresholds were defined as the point where the cumulative percentage reached ≥50%. We used logistic regression to analyze physician characteristics associated with reporting no age threshold versus any age threshold.

## 3 Results

### 3.1 Physician characteristics

Overall, 503 physicians, comprising 347 oncologists (69%) and 156 urologists (31%), completed the survey; most were male (72%), had practiced for >10 yr (69%), treated from five to 19 patients with mUC per month (58%), and practiced in a public teaching (40%) or nonteaching hospital (24%; Table 1). Considerable differences in physician characteristics were seen across the five countries, except for age. Most physicians in Spain and the UK practiced in public teaching hospitals, while physicians from Italy and France had more equal distributions across hospital-based practice settings. Few physicians practiced in non–hospital-based settings (public and private offices or specialist cancer centers), except for 24% of physicians in Germany who practiced in a public office. During the previous 6 mo, physicians in Italy and the UK had seen the most patients with mUC (median, 50).

**Table 1:** Physician demographic and practice characteristics

	Full sample (*N* = 503)	Germany (*n* = 101)	Spain (*n* = 102)	France (*n* = 100)	Italy (*n* = 100)	UK (*n* = 100)	*p* value
Sex, *n* (%)							<0.001
Female	112 (22)	19 (19)	47 (46)	13 (13)	18 (18)	15 (15)	
Male	362 (72)	79 (78)	49 (48)	83 (83)	76 (76)	75 (75)	
I prefer not to answer	29 (5.8)	3 (3.0)	6 (5.9)	4 (4.0)	6 (6.0)	10 (10)	
Age (yr)	(*n* = 425)	(*n* = 90)	(*n* = 82)	(*n* = 83)	(*n* = 92)	(*n* = 78)	0.6
Median (IQR)	44 (40–49)	44 (41–51)	43 (39–47)	45 (40–49)	44 (40–48)	44 (39–49)	
Time in practice (yr), *n* (%)							0.083
3–6	23 (4.6)	3 (3.0)	3 (2.9)	9 (9.0)	3 (3.0)	5 (5.0)	
7–10	134 (27)	22 (22)	24 (24)	31 (31)	39 (39)	18 (18)	
11–14	167 (33)	35 (35)	39 (38)	27 (27)	28 (28)	38 (38)	
15–18	94 (19)	20 (20)	19 (19)	16 (16)	14 (14)	25 (25)	
>18	85 (17)	21 (21)	17 (17)	17 (17)	16 (16)	14 (14)	
Primary specialty, *n* (%)							0.007
Oncology	347 (69)	55 (54)	72 (71)	74 (74)	77 (77)	69 (69)	
Urology	156 (31)	46 (46)	30 (29)	26 (26)	23 (23)	31 (31)	
Time since graduated medical school (yr)	(*n* = 400)	(*n* = 81)	(*n* = 82)	(*n* = 82)	(*n* = 82)	(*n* = 73)	0.4
Mean (SD)	17 (8.3)	18 (8.7)	17 (7.2)	17 (8.9)	17 (9.4)	18 (7.4)	
Median (IQR)	15 (12–22)	14 (12–22)	15 (13–20)	15 (12–20)	14 (10–23)	16 (13–21)	
Time spent providing direct patient care, *n* (%)							<0.001
50–74%	77 (15)	13 (13)	7 (6.9)	26 (26)	26 (26)	5 (5.0)	
75–100%	426 (85)	88 (87)	95 (93)	74 (74)	74 (74)	95 (95)	
Practice type, *n* (%)							<0.001
Public teaching hospital	203 (40)	33 (33)	59 (58)	32 (32)	24 (24)	55 (55)	
Public nonteaching hospital	121 (24)	25 (25)	21 (21)	19 (19)	32 (32)	24 (24)	
Private hospital	102 (20)	7 (6.9)	20 (20)	37 (37)	24 (24)	14 (14)	
Public office	36 (7.2)	24 (24)	0	3 (3.0)	5 (5.0)	4 (4.0)	
Private office	18 (3.6)	5 (5.0)	0	4 (4.0)	9 (9.0)	0	
Specialist cancer center	23 (4.6)	7 (6.9)	2 (2.0)	5 (5.0)	6 (6.0)	3 (3.0)	
Hospital size, *n* (%)^a^	(*n* = 426)	(*n* = 65)	(*n* = 100)	(*n* = 88)	(*n* = 80)	(*n* = 93)	<0.001
Large (≥500 beds)	175 (41)	33 (51)	36 (36)	35 (40)	19 (24)	52 (56)	
Medium (100–499 beds)	234 (55)	29 (45)	63 (63)	48 (55)	55 (69)	39 (42)	
Small (<100 beds)	17 (4.0)	3 (4.6)	1 (1.0)	5 (5.7)	6 (7.5)	2 (2.2)	
Average no. of patients with mUC treated per month, *n* (%)							<0.001
2–4	76 (15)	12 (12)	19 (19)	11 (11)	12 (12)	22 (22)	
5–10	153 (30)	44 (44)	30 (29)	20 (20)	29 (29)	30 (30)	
11–19	141 (28)	25 (25)	17 (17)	30 (30)	49 (49)	20 (20)	
20–50	99 (20)	19 (19)	27 (26)	30 (30)	8 (8.0)	15 (15)	
>50	34 (6.8)	1 (1.0)	9 (8.8)	9 (9.0)	2 (2.0)	13 (13)	
No. of patients with mUC seen in the past 6 mo							0.002
Median (IQR)	44 (30–70)	40 (22–55)	37 (25–55)	45 (30–90)	50 (33–90)	50 (33–70)	

IQR = interquartile range; mUC = metastatic urothelial carcinoma; SD = standard deviation.

a Applicable only to participants who practice in a public teaching hospital, public nonteaching hospital, or private hospital.

### 3.2 Proportion of patients eligible for platinum-based chemotherapy

**Fig. 1:**
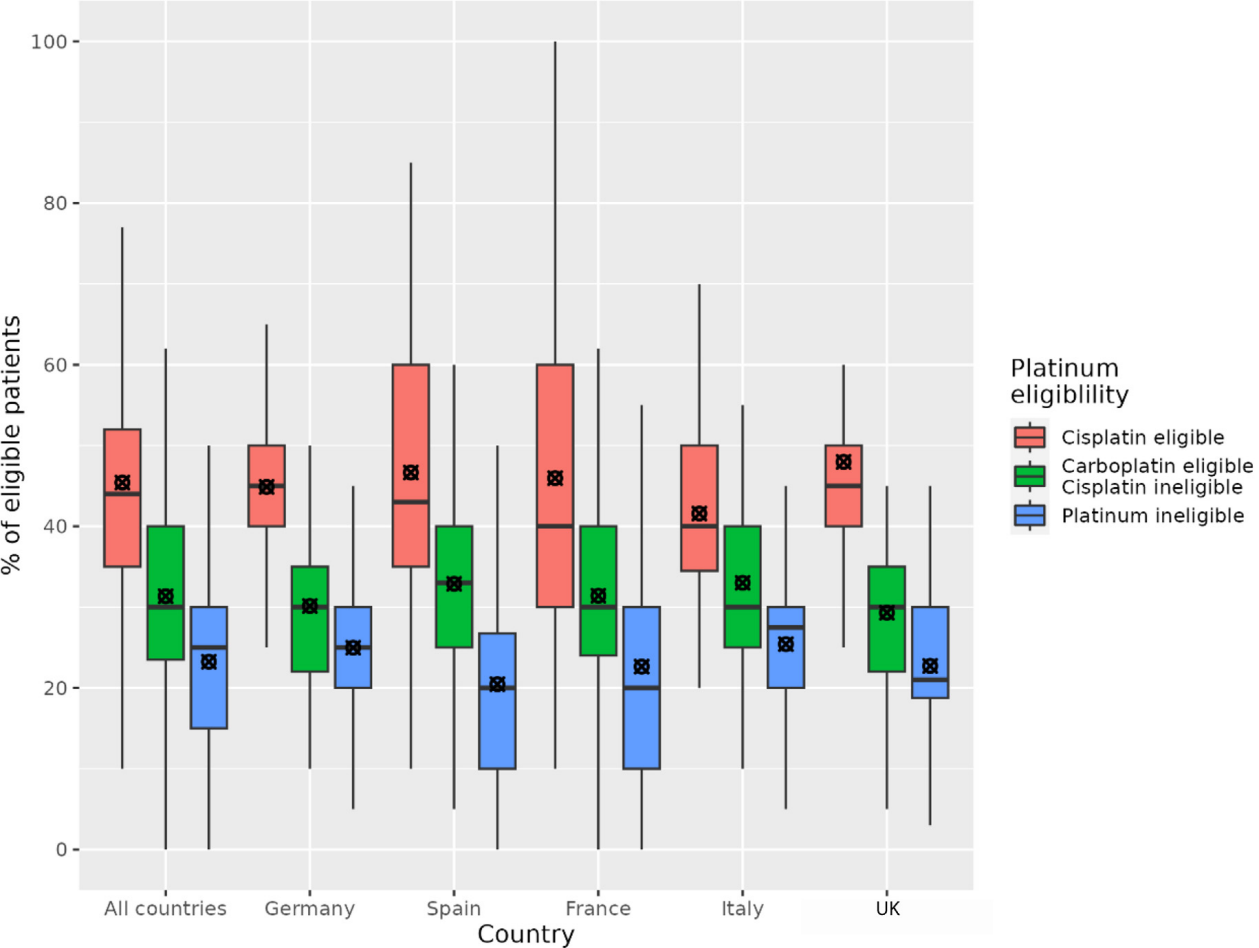
Percentage of patients with metastatic urothelial carcinoma whom physicians reported as cisplatin eligible, carboplatin eligible only, or platinum ineligible for first-line chemotherapy. Box and whisker plots show the median (horizontal black line), mean (circle with ×), interquartile range (top to bottom of box), and 2.5th and 97.5th percentile data range (whiskers).

Physicians reported that most patients with mUC who received 1L systemic anticancer treatment were eligible for either cisplatin (mean, 45.4%) or carboplatin (mean, 31.3%; Fig. 1). Across countries, percentages of patients reported as eligible for cisplatin ranged from 41.6% (Italy) to 48.0% (UK), while percentages eligible for carboplatin ranged from 29.3% (UK) to 33.0% (Italy; Supplementary Table 2). Physicians reported that a mean of 76.8% of patients were platinum eligible, ranging from 74.6% (Italy) to 79.5% (Spain).

### 3.3 Platinum-based chemotherapy decision-making factors

**Fig. 2:**
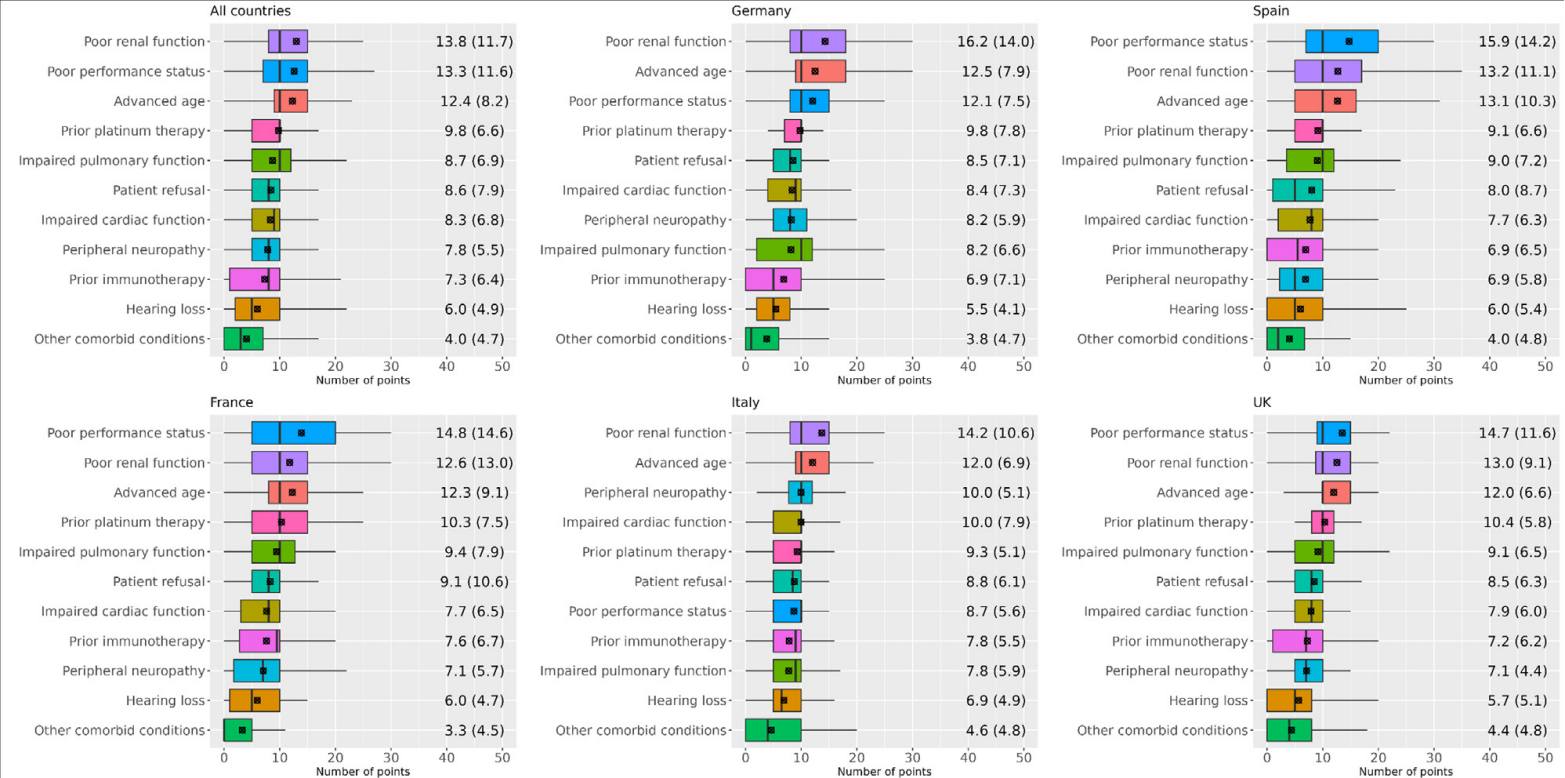
Patient factors that physicians considered in their decision to treat patients with metastatic urothelial carcinoma with first-line platinum-based (cisplatin or carboplatin) chemotherapy. Characteristics are listed in order of importance from top to bottom based on the mean number of points. Box and whisker plots show the median (horizontal black line), mean (circle with ×), interquartile range (top to bottom of box), and 2.5th and 97.5th percentile data range (whiskers).

The top factors physicians considered when deciding whether to treat with 1L platinum-based chemotherapy were poor renal function (mean, 13.8 points allocated out of 100), poor PS (mean, 13.3), and advanced age (mean, 12.4; Fig. 2). Based on median scores, physicians ranked these factors equally with prior platinum therapy and impaired pulmonary function (median, 10). Rankings were similar across countries, except for poor PS and peripheral neuropathy (Supplementary Table 3), which were ranked as the third and seventh most important factors in Italy, respectively, compared with eighth and second in the overall sample.

### 3.4 Platinum-based chemotherapy ineligibility criteria

Statistically significant differences were noted in the distribution of thresholds used to define platinum ineligibility between the full European sample and prior US survey results for CrCl overall, CrCl in patients with ECOG PS 2, advanced age, and NYHA heart failure class (Table 2). For peripheral neuropathy, a difference in thresholds was seen between the full European sample and 2019 US results (*p* = 0.038) but not 2022 US results. No statistically significant differences for ECOG PS were seen. Significant differences were observed across European countries for all criteria except ECOG PS and peripheral neuropathy (Supplementary Table 4).

**Table 2:** Physician self-reported thresholds used to define platinum ineligibility for patients with mUC receiving 1L therapy: comparison of the full sample results with prior US results

	Full sample (*N* = 503)	2019 US survey [19] (*N* = 43)	*p* value^a^	2022 US survey [20] (*N* = 60)	*p* value^a^
	*n* (%)	*n* (%)		*n* (%)	
CrCl threshold to define platinum ineligibility			<0.001		<0.001
None	7 (1.4)	NR^b^		NR^b^	
<30 ml/min	175 (35)	25 (58)		41 (68)	
<25 ml/min	144 (29)	2 (4.7)		1 (1.7)	
<20 ml/min	134 (27)	1 (2.3)		5 (8.3)	
<15 ml/min	28 (5.6)	4 (9.3)		6 (10)	
<10 ml/min	3 (0.6)	1 (2.3)		2 (3.3)	
Other	12 (2.4)	10 (23)		5 (8.3)	
CrCl cutoff to define platinum ineligibility for a patient with ECOG PS 2			<0.001		<0.001
None	6 (1.2)	NR^b^		NR^b^	
<60 ml/min	50 (9.9)	6 (14)		2 (3.3)	
<50 ml/min	101 (20)	8 (19)		7 (12)	
<40 ml/min	114 (23)	6 (14)		10 (17)	
<30 ml/min	162 (32)	15 (35)		29 (48)	
<20 ml/min	64 (13)	1 (2.3)		4 (6.7)	
<10 ml/min	4 (0.8)	2 (4.7)		1 (1.7)	
Other	2 (0.4)	5 (12)		7 (12)	
Age threshold to define platinum ineligibility			<0.001		
None	59 (12)	31 (72)		NA	
>70 yr	48 (9.5)	2 (4.7)		NA	
>75 yr	121 (24)	0		NA	
>80 yr	167 (33)	3 (7.0)		NA	
>85 yr	77 (15)	2 (4.7)		NA	
>90 yr	31 (6.2)	4 (9.3)		NA	
Other	0	1 (2.3)		NA	
ECOG PS threshold to define platinum ineligibility					
None	4 (0.8)	NR^b^		NR^b^	
ECOG PS ≥0	3 (0.6)	NR^b^		NR^b^	
ECOG PS ≥1	40 (8.0)	NR^b^		NR^b^	
ECOG PS ≥2	204 (41)	11 (26)	0.073^c^	19 (32)	0.2^c^
ECOG PS ≥3	228 (45)	32 (74)		41 (68)	
ECOG PS 4	24 (4.8)	NR^b^		NR^b^	
NYHA heart failure class to define platinum ineligibility			0.028		0.001
None	23 (4.6)	5 (12)		3 (5.0)	
Class I	7 (1.4)	0^a^		0^a^	
Class II	123 (24)	3 (7.0)		2 (3.3)	
Class III	294 (58)	29 (67)		48 (80)	
Class IV	56 (11)	6 (14)		7 (12)	
Grade of peripheral neuropathy to define platinum ineligibility					
None	16 (3.2)	0^a^		0^a^	
Grade ≥1	16 (3.2)	0^a^		0^a^	
Grade ≥2	223 (44)	12 (28)	0.038^c^	32 (53)	0.2^c^
Grade ≥3	210 (42)	30 (70)		0^a^	
Grade 4	37 (7.4)	0^a^		0^a^	
Other	1 (0.2)	1 (2.3)		28 (47)	

CrCl = creatinine clearance; ECOG PS = Eastern Cooperative Oncology Group performance status; 1L = first line; mUC = metastatic urothelial carcinoma; NA = not applicable (the question was not included in the survey); NHYA = New York Heart Association; NR = not reported.

a Differences between the full sample and previous US results (2019 and 2022) for each criterion were assessed using Fisher’s exact test for count data with simulated *p* value (based on 2000 replicates).

b Only publicly available data were used. For response options where no data were reported in the respective publications, it was assumed that the question level was available and that there were zero respondents.

c Only percentages in nonzero categories were included in the estimation of the *p* values.

**Fig. 3:**
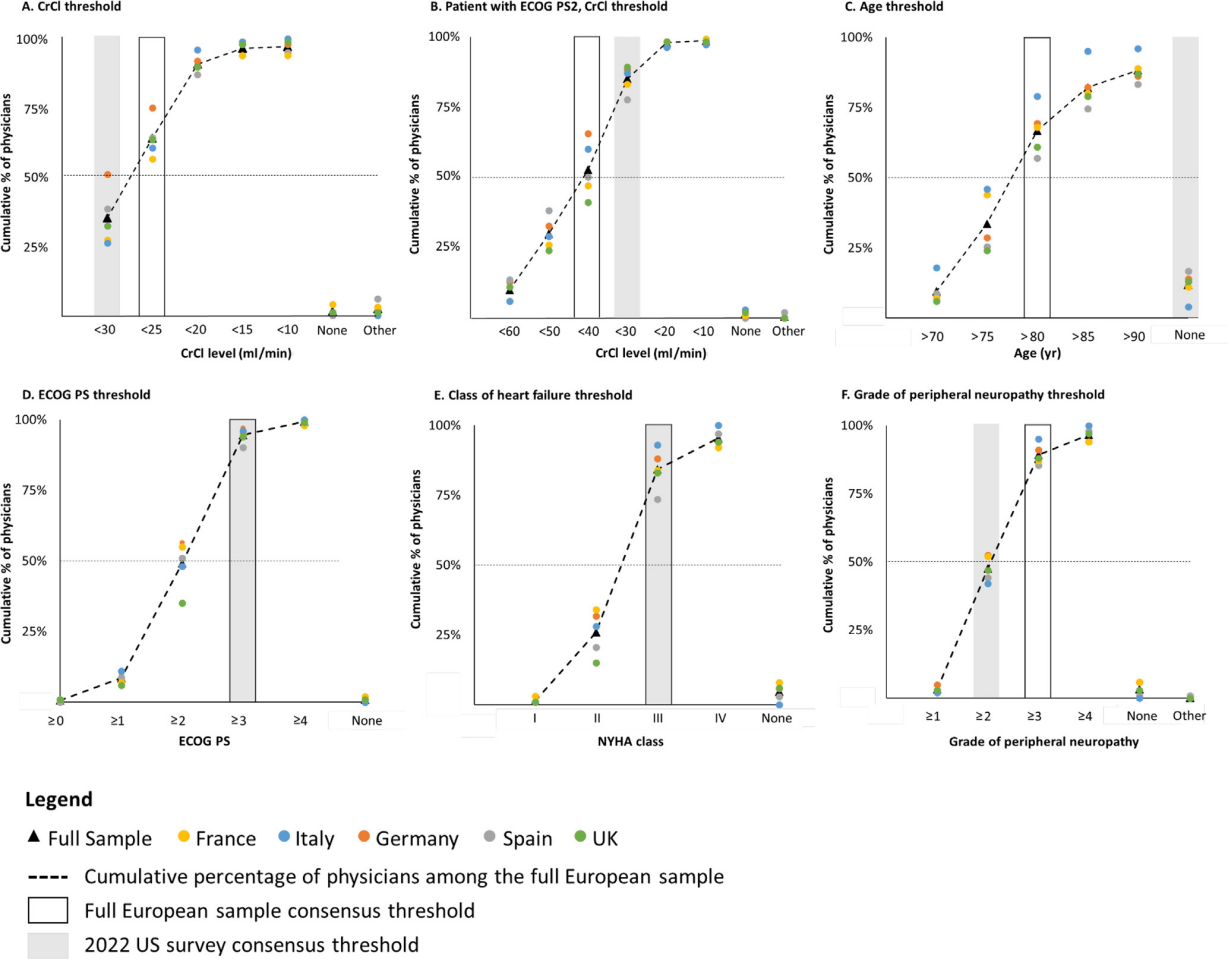
Cumulative percentage of physicians who would not treat patients with metastatic urothelial carcinoma with first-line platinum-based chemotherapy at each threshold. The consensus threshold was defined as the threshold at which the cumulative percentage reached ≥50%. CrCl = creatinine clearance; ECOG PS = Eastern Cooperative Oncology Group performance status; NHYA = New York Heart Association.

Consensus thresholds in the full sample were broadly consistent with thresholds from the 2022 US survey, except for advanced age (Fig. 3). Advanced age was not considered to be a criterion for platinum ineligibility in the US surveys; however, in this European survey, 67% of physicians would not treat patients aged >80 yr with platinum-based chemotherapy. The full European sample reached consensus for a CrCl threshold of <25 ml/min overall or <40 ml/min for patients with ECOG PS 2, compared with <30 ml/min overall in the US survey. The full European sample and the 2022 US sample were aligned on thresholds for ECOG PS (≥3) and NYHA heart failure (class III); for NYHA, this consensus was reached in all five European countries. The full sample reached consensus for peripheral neuropathy at grade ≥3 compared with grade ≥2 in the US 2022 survey.

### 3.5 Physician characteristics associated with using an age threshold to determine platinum ineligibility

The majority of physicians reported using an age threshold to define platinum ineligibility (*n* = 444; 88%) versus no age threshold (*n* = 59; 12%; Supplementary Table 5). After adjusting for baseline characteristics, physicians were more likely to use an age threshold if they were Italian versus German (adjusted odds ratio, 0.22 [95% confidence interval, 0.06–0.73]), practiced in a public nonteaching hospital (0.28 [0.10–0.73]) or in public or private office settings versus a private hospital (0.14 [0.03–0.57]), and treated <20 patients with mUC per month (from two to ten patients: 0.36 [0.17–0.74]; 11–19 patients: 0.25 [0.09–0.62]) versus >20 patients.

## 4 Discussion

In this study, practicing oncologists and urologists in five European countries considered most patients with mUC receiving 1L treatment to be eligible for platinum-based chemotherapy. The top factors considered by European physicians to determine platinum ineligibility were consistent with those reported in the 2019 and 2022 US-based surveys and EAU guidelines [7,19,20]. Thresholds for these criteria were broadly consistent with the US thresholds and EAU guidelines, except for age. Most US physicians reported no age threshold for platinum ineligibility, compared with 12% of European physicians. These results suggest broad alignment between European and US physicians regarding platinum ineligibility criteria in patients with mUC, with some notable caveats.

Physicians in our survey study reported that ∼25% of patients with mUC receiving 1L treatment are platinum ineligible. However, a recent real-world study in the same European countries found that 13% of patients receiving 1L treatment were platinum ineligible [22], suggesting that physicians in our survey may inappropriately define some patients as platinum ineligible. Real-world studies of 1L treatment patterns in mUC have shown variations in percentages of patients receiving a platinum-based regimen [[13], [14], [15], [16]], underscoring the need for a globally standardized definition of platinum ineligibility.

Despite variations between countries, the criteria that European physicians used to define platinum ineligibility were broadly consistent with previous results, except for age [19,20]. We observed strong agreement that patients with ECOG PS ≥3 and NYHA heart failure class III should be classified as platinum ineligible. Similar to the 2022 US survey, European physicians were evenly split between peripheral neuropathy thresholds of grade ≥2 (44%) and grade ≥3 (42%); the consensus threshold was reached at grade ≥3 in our survey, compared with grades ≥2 and ≥3 in the 2022 and 2019 US surveys, respectively. Previous studies have shown that renal impairment is one of the most important platinum-ineligibility criteria [22,23]. US studies reported that the CrCl threshold for all patients, regardless of ECOG PS, should be <30 ml/min. However, the European consensus threshold was <25 ml/min overall and <40 ml/min in patients with ECOG PS 2. Physicians in France and the UK were in alignment with those participating in previous US surveys for patients with ECOG PS 2 (CrCl threshold <30 ml/min). Our sample’s responses were also broadly consistent with the CrCl criteria used by a previous retrospective study of outcomes in platinum-ineligible patients (<30 ml/min overall and 30–59 ml/min in patients with ECOG PS 2) [23].

Differences were noted regarding the use of chronological age to define platinum-ineligible patients. US-based studies reported that age was not a criterion for platinum ineligibility. However, in our European sample, most respondents selected >80 yr (33%) or >75 yr (24%) as the age threshold to define platinum ineligibility, with 12% selecting no age threshold. Physicians were more likely to have an age threshold if they were from Italy, practiced in a public nonteaching hospital or a public or private office setting, and treated <20 patients with mUC per month. While age has been noted as an important factor impacting treatment choice [14,22,23], including a previous study that found age as one of the most common reasons that patients were deemed platinum ineligible [22], clinical experts have emphasized the distinction between chronological and functional age [24,25]. This distinction is critical because mUC occurs predominantly in older adults (median age at diagnosis, 69 yr in men and 71 yr in women) [26]. According to clinical experts and prior US-based surveys, patients should not be denied platinum-based chemotherapy based on chronological age alone [19,20,24]. European physicians who use a chronological age threshold may be inappropriately excluding patients from receiving platinum-based chemotherapy; this criterion could be one of the driving factors of systemic therapy underuse in mUC [17,27].

This study has several limitations. First, each threshold question was asked separately; however, the decision to treat a patient with platinum-based chemotherapy may involve a combination of factors. Physicians may use clinical criteria and patient characteristics, such as frailty or comorbidity burden, for decision-making that were not included in this survey. Further, the use of clinical criteria may be influenced by local institutional guidelines, which were not assessed in this study. As participation was voluntary, recruitment and self-selection bias may be present. All information was self-reported; thus, the potential exists for a response bias. Lastly, the use of a convenience sample may limit the generalizability of findings. Despite these limitations, our study provides valuable insights into the 1L treatment decision-making process for mUC across five European countries.

## 5 Conclusions

Practicing oncologists and urologists in Europe considered most patients with mUC receiving 1L treatment to be eligible for platinum-based chemotherapy. The criteria used to determine platinum ineligibility were broadly consistent with the EAU guidelines and prior US-based studies, except for age. A lack of a uniformly applied definition of platinum ineligibility may contribute to variations in quality of care and patient outcomes across practice settings. Implementation of standardized criteria for determining platinum ineligibility could support decision-making at the point of care, reduce variations in treatment, help define criteria for clinical trial eligibility, and improve patient outcomes.

  ***Author contributions:*** Shilpa Gupta had full access to all the data in the study and takes responsibility for the integrity of the data and the accuracy of the data analysis.

  *Study concept and design*: Gupta, Kearney, Kirker, Bharmal, Guenther, Costa.

*Acquisition of data*: Panattoni, Land, Flottemesch, Sullivan.

*Analysis and interpretation of data*: All authors.

*Drafting of the manuscript*: All authors.

*Critical revision of the manuscript for important intellectual content*: All authors.

*Statistical analysis*: Panattoni, Land, Flottemesch, Sullivan.

*Obtaining funding*: Kearney.

*Administrative, technical, or material support*: Kearney.

*Supervision*: Gupta, Kearney, Bharmal.

*Other*: None.

  ***Financial disclosures:*** Shilpa Gupta certifies that all conflicts of interest, including specific financial interests and relationships and affiliations relevant to the subject matter or materials discussed in the manuscript (eg, employment/affiliation, grants or funding, consultancies, honoraria, stock ownership or options, expert testimony, royalties, or patents filed, received, or pending), are the following: Shilpa Gupta has provided speaker services for Bristol Myers Squibb, Exelixis, and Janssen; has received honoraria from AstraZeneca, Bristol Myers Squibb, Exelixis, MSD, and Seagen; and has received research funding from Bristol Myers Squibb, Merck, Pfizer, and Seagen. Thomas Powles reports a consulting or advisory role for Astellas Pharma, AstraZeneca, Bristol Myers Squibb, Eisai, Exelixis, Incyte, Ipsen, Johnson & Johnson, Merck, MSD, Novartis, Pfizer, Roche, and Seagen; has received research funding from Astellas Pharma, AstraZeneca, Bristol Myers Squibb, Eisai, Exelixis, Ipsen, Johnson & Johnson, Merck, MSD, Novartis, Pfizer, Roche, and Seagen; and has received travel and accommodation expenses from AstraZeneca, Ipsen, MSD, Pfizer, and Roche. Mairead Kearney is an employee of Merck and holds stock and other ownership interests with Merck, Novartis, and UCB. Silke Guenther was an employee of Merck when the study was conducted. Laura Panattoni was an employee of PRECISIONheor when the study was conducted. Natalie Land and Thomas Flottemesch are employees of PRECISIONheor, a research consultancy that provides health economics and outcomes research services to life sciences companies; PRECISIONheor received payment from the study funder for the work described. Patrick Sullivan was an employee of PRECISIONheor when the study was conducted. Murtuza Bharmal was an employee of EMD Serono, Research & Development Institute, Inc., Billerica, MA, USA, an affiliate of Merck KGaA, when the study was conducted. Melissa Kirker and Nuno Costa are employees of Pfizer. Enrique Grande has received honoraria for speaker engagements, advisory roles, or funding of continuous medical education from Advanced Accelerator Applications, Adium, Amgen, Angelini, Astellas, AstraZeneca, Bayer, Blueprint, Bristol Myers Squibb, Caris Life Sciences, Celgene, Clovis Oncology, Eisai, EUSA Pharma, Genetracer, Guardant Health, HRA Pharma, Ipsen, ITM Radiopharma, Janssen, Lexicon, Lilly, Merck, MSD, Nanostring Technologies, Natera, Novartis, OncoDNA (Biosequence), Palex, PharmaMar, Pierre Fabre, Pfizer, Roche, Sanofi-Genzyme, Servier, Taiho, and Thermo Fisher Scientific; and has also received research grants from AstraZeneca, Astellas, Lexicon Pharmaceuticals, Merck, and Pfizer.

  ***Funding/Support and role of the sponsor:*** This study was funded by Merck (CrossRef Funder ID: 10.13039/100009945) and was previously conducted under an alliance between Merck and Pfizer.

  ***Data sharing statement:*** Any requests for data by qualified scientific and medical researchers for legitimate research purposes will be subject to Merck’s data sharing policy. All requests should be submitted in writing to Merck’s data sharing portal (https://www.merckgroup.com/en/research/our-approach-to-research-and-development/healthcare/clinical-trials/commitment-responsible-data-sharing.html). When Merck has a coresearch, codevelopment, or comarketing or copromotion agreement, or when the product has been out-licensed, the responsibility for disclosure might be dependent on the agreement between parties. Under these circumstances, Merck will endeavor to gain agreement to share data in response to requests.

  ***Acknowledgments:*** The authors thank all the participants of this study. Editorial support was provided by Nucleus Global and funded by Merck.
