# Supplementary material for: Criteria Used to Determine Platinum Eligibility in Patients with Metastatic Urothelial Carcinoma: Results of a Physician Survey in Five European Countries
Source: Eur Urol Open Sci. 2025 Sep 23;81:28–36. doi: 10.1016/j.euros.2025.08.009 (PMC12495075; doi:10.1016/j.euros.2025.08.009)
Supplement: Supplementary Data 1 [file mmc1.docx]

**Supplementary Table 1.** Survey items (English version)

| 1. Over the past 6 months, what percentage of your mUC patients receiving 1L systemic anticancer treatment are:   *(Total must sum to 100%)* |
| --- |
| Cisplatin-eligible ____% |
| Cisplatin-ineligible, but carboplatin-eligible ___% |
| Platinum-ineligible (ie, ineligible for cisplatin and carboplatin) ___% |
| 1. Please distribute 100 points across the characteristics below you consider when determining whether to treat an mUC patient with 1L platinum-based chemotherapy? You can use any number between 0 to 100 for each characteristic to arrive at a total of 100 points, where 0 is the lowest importance and 100 is the highest importance.   *(Total must sum to 100)* |
| Impaired pulmonary function ____ |
| Advanced age ____ |
| Prior platinum therapy (neoadjuvant/adjuvant) ____ |
| Prior immunotherapy (neoadjuvant/adjuvant) ____ |
| Poor performance status ____ |
| Patient refusal ____ |
| Poor renal function ____ |
| Peripheral neuropathy ____ |
| Hearing loss ____ |
| Impaired cardiac function ____ |
| Other comorbid conditions ____ |
| 1. What threshold creatinine clearance do you use to define "platinum ineligibility" (ie, ineligible for cisplatin and carboplatin)? |
| - None |
| - <30 mL/min |
| - <25 mL/min |
| - <20 mL/min |
| - <15 mL/min |
| - <10 mL/min |
| - Other, please specify: _______ |
| 1. What age threshold do you use to define "platinum ineligibility" (ie, ineligible for cisplatin and carboplatin)? |
| - None |
| - >70 years |
| - >75 years |
| - >80 years |
| - >85 years |
| - >90 years |
| - Other, please specify:_______ |
| 1. What ECOG performance status threshold do you use to define "platinum ineligibility" (ie, ineligible for cisplatin and carboplatin)? |
| - None |
| - ECOG ≥0 |
| - ECOG ≥1 |
| - ECOG ≥2 |
| - ECOG ≥3 |
| - ECOG ≥4 |
| - Other, please specify:_____ |
| 1. In a patient with ECOG performance status 2, what creatinine clearance cut-off do you use to define "platinum ineligibility" (ie, ineligible for cisplatin and carboplatin)? |
| - None |
| - <60 mL/min |
| - <50 mL/min |
| - <40 mL/min |
| - <30 mL/min |
| - <20 mL/min |
| - <10 mL/min |
| - Other, please specify:_____ |
| 1. What class of Heart Failure do you use to define "platinum ineligibility" (ie, ineligible for cisplatin and carboplatin)? |
| - None |
| - NYHA Class I |
| - NYHA Class II |
| - NYHA Class III |
| - NYHA Class IV |
| - Other, please specify:_____ |
| 1. What grade of peripheral neuropathy do you use to define "platinum ineligibility"(ie, ineligible for cisplatin and carboplatin)? |
| - None |
| - ≥Grade 1 |
| - ≥Grade 2 |
| - ≥Grade 3 |
| - ≥Grade 4 |
| - Other, please specify:_____ |

1L = first line; ECOG = Eastern Cooperative Oncology Group; mUC = metastatic urothelial carcinoma; NHYA = New York Heart Association.

**Supplementary Table 2.** Percentage of patients with mUC physicians reported as cisplatin eligible, carboplatin eligible only, or platinum ineligible for 1L chemotherapy

|  | **Full sample**  **(*N =* 503)** | **Germany**  **(*n =* 101)** | **Spain**  **(*n =* 102)** | **France**  **(*n =* 100)** | **Italy**  **(*n =* 100)** | **UK**  **(*n =* 100)** | |
| --- | --- | --- | --- | --- | --- | --- | --- |
| **Cisplatin eligible** |  |  |  |  |  |  | |
| Median (IQR), % | 44 (35-52) | 45 (40-50) | 43 (35-60) | 40 (30-60) | 40 (34-50) | 45 (40-50) | |
| Mean (SD), % | 45.4 (14.9) | 44.9 (13.9) | 46.7 (15.1) | 46.0 (17.9) | 41.6 (11.2) | 48.0 (15.4) | |
| **Carboplatin eligible, cisplatin ineligible** |  |  |  |  |  |  | |
| Median (IQR), % | 30 (24-40) | 30 (22-35) | 33 (25-40) | 30 (24-40) | 30 (25-40) | 30 (22-35) | |
| Mean (SD), % | 31.3 (12.5) | 30.1 (14.2) | 32.9 (12.9) | 31.4 (13.6) | 33.0 (11.0) | 29.3 (10.5) | |
| **Platinum ineligible** |  |  |  |  |  |  | |
| Median (IQR), % | 25 (15-30) | 25 (20-30) | 20 (10-27) | 20 (10-30) | 28 (20-30) | 21 (19-30) | |
| Mean (SD), % | 23.2 (12.2) | 25.0 (12.1) | 20.5 (11.1) | 22.6 (13.9) | 25.4 (11.9) | 22.7 (11.1) | |
| 1L = first line; IQR = interquartile range; mUC = metastatic urothelial carcinoma; SD = standard deviation. | | | | | | |  |

**Supplementary Table 3.** Patient factors physicians considered in their decision to treat patients with mUC with 1L platinum-based (cisplatin or carboplatin) chemotherapy

|  | **Full sample**  **(*N =* 503)** | **Germany**  **(*n =* 101)** | **Spain**  **(*n =* 102)** | **France**  **(*n =* 100)** | **Italy**  **(*n =* 100)** | **UK**  **(*n =* 100)** |
| --- | --- | --- | --- | --- | --- | --- |
| **Impaired pulmonary function** |  |  |  |  |  |  |
| Median (IQR) | 10 (5-12) | 10 (2-12) | 10 (4-12) | 10 (5-13) | 9 (5-10) | 10 (5-12) |
| Mean (SD) | 8.7 (6.9) | 8.2 (6.6) | 9.0 (7.2) | 9.4 (7.9) | 7.8 (5.9) | 9.1 (6.5) |
| **Advanced age** |  |  |  |  |  |  |
| Median (IQR) | 10 (9-16) | 10 (9-18) | 10 (6-19) | 10 (8-15) | 10 (9-15) | 13 (10-15) |
| Mean (SD) | 12.4 (8.2) | 12.5 (7.9) | 13.1 (10.3) | 12.3 (9.1) | 12.0 (6.9) | 12.0 (6.6) |
| **Prior platinum therapy** |  |  |  |  |  |  |
| Median (IQR) | 10 (5-10) | 10 (7-10) | 10 (5-10) | 10 (5-15) | 10 (5-10) | 10 (8-12) |
| Mean (SD) | 9.8 (6.6) | 9.8 (7.8) | 9.1 (6.6) | 10.3 (7.5) | 9.3 (5.1) | 10.4 (5.8) |
| **Prior immunotherapy** |  |  |  |  |  |  |
| Median (IQR) | 8 (1-10) | 5 (0-10) | 6 (0-10) | 10 (3-10) | 9 (5-10) | 7 (0-10) |
| Mean (SD) | 7.3 (6.4) | 6.9 (7.1) | 6.9 (6.5) | 7.6 (6.7) | 7.8 (5.5) | 7.2 (6.2) |
| **Poor PS** |  |  |  |  |  |  |
| Median (IQR) | 10 (8-15) | 10 (8-15) | 10 (7-20) | 10 (5-20) | 10 (5-10) | 11 (10-20) |
| Mean (SD) | 13.3 (11.6) | 12.1 (7.5) | 15.9 (14.2) | 14.8 (14.6) | 8.7 (5.6) | 14.7 (11.6) |
| **Patient refusal** |  |  |  |  |  |  |
| Median (IQR) | 8 (5-10) | 8 (5-10) | 5 (1-10) | 8 (5-10) | 8 (5-10) | 8 (5-10) |
| Mean (SD) | 8.6 (7.9) | 8.5 (7.1) | 8.0 (8.7) | 9.1 (10.6) | 8.8 (6.1) | 8.5 (6.3) |
| **Poor renal function** |  |  |  |  |  |  |
| Median (IQR) | 10 (8-16) | 10 (8-20) | 10 (5-18) | 10 (5-15) | 10 (8-15) | 10 (10-15) |
| Mean (SD) | 13.8 (11.7) | 16.2 (14.0) | 13.2 (11.1) | 12.6 (13.0) | 14.2 (10.6) | 13.0 (9.1) |
| **Peripheral neuropathy** |  |  |  |  |  |  |
| Median (IQR) | 8 (5-10) | 8 (5-11) | 5 (2-10) | 7 (2-10) | 10 (8-12) | 7 (5-10) |
| Mean (SD) | 7.8 (5.5) | 8.2 (5.9) | 6.9 (5.8) | 7.1 (5.7) | 10.0 (5.1) | 7.1 (4.4) |
| **Hearing loss** |  |  |  |  |  |  |
| Median (IQR) | 5 (2-10) | 5 (2-8) | 5 (0-10) | 5 (1-10) | 7 (5-10) | 5 (3-8) |
| Mean (SD) | 6.0 (4.9) | 5.5 (4.1) | 6.0 (5.4) | 6.0 (4.7) | 6.9 (4.9) | 5.7 (5.1) |
| **Impaired cardiac function** |  |  |  |  |  |  |
| Median (IQR) | 9 (5-10) | 9 (4-10) | 8 (2-10) | 8 (3-10) | 10 (5-10) | 8 (5-10) |
| Mean (SD) | 8.3 (6.8) | 8.4 (7.3) | 7.7 (6.3) | 7.7 (6.5) | 10.0 (7.9) | 7.9 (6.0) |
| **Other comorbid conditions** |  |  |  |  |  |  |
| Median (IQR) | 3 (0-7) | 1 (0-6) | 2 (0-7) | 0 (0-5) | 4 (0-10) | 4 (0-8) |
| Mean (SD) | 4.0 (4.7) | 3.8 (4.7) | 4.0 (4.8) | 3.3 (4.5) | 4.6 (4.8) | 4.4 (4.8) |

1L = first line; IQR = interquartile range; mUC = metastatic urothelial carcinoma; PS = performance status; SD = standard deviation.

Physicians were asked to allocate 100 points across the 11 clinical factors shown in the table. Physicians were required to allocate between 0 and 100 points to each factor (0, lowest importance; 100, highest importance) and to allocate all 100 points. Median and mean points allocated are shown.

**Supplementary Table 4.** Physician self-reported thresholds used to define platinum ineligibility for patients with mUC receiving 1L therapy

|  | **Full sample**  **(*N =* 503)** | **Germany**  **(*n =* 101)** | **Spain**  **(*n =* 102)** | **France**  **(*n =* 100)** | **Italy**  **(*n =* 100)** | **UK**  **(*n =* 100)** |  |
| --- | --- | --- | --- | --- | --- | --- | --- |
|  | ***n (%)*** | ***n (%)*** | ***n (%)*** | ***n (%)*** | ***n (%)*** | ***n (%)*** | ***p* Value** |
| **CrCl threshold to define platinum ineligibility** |  |  |  |  |  |  | 0.006 |
| None | 7 (1.4) | 1 (1.0) | 0 | 4 (4.0) | 1 (1.0) | 1 (1.0) |  |
| <30 mL/min | 175 (35) | 51 (50) | 39 (38) | 27 (27) | 26 (26) | 32 (32) |  |
| <25 mL/min | 144 (29) | 24 (24) | 26 (25) | 29 (29) | 34 (34) | 31 (31) |  |
| <20 mL/min | 134 (27) | 17 (17) | 23 (23) | 33 (33) | 35 (35) | 26 (26) |  |
| <15 mL/min | 28 (5.6) | 6 (5.9) | 7 (6.9) | 4 (4.0) | 3 (3.0) | 8 (8.0) |  |
| <10 mL/min | 3 (0.6) | 0 | 1 (1.0) | 0 | 1 (1.0) | 1 (1.0) |  |
| Other | 12 (2.4) | 2 (2.0) | 6 (5.9) | 3 (3.0) | 0 | 1 (1.0) |  |
| **CrCl cutoff to define platinum ineligibility for a patient with ECOG PS 2** |  |  |  |  |  |  | <0.001 |
| None | 6 (1.2) | 0 | 0 | 1 (1.0) | 3 (3.0) | 2 (2.0) |  |
| <60 mL/min | 50 (9.9) | 13 (13) | 14 (14) | 6 (6.0) | 6 (6.0) | 11 (11) |  |
| <50 mL/min | 101 (20) | 20 (20) | 25 (25) | 20 (20) | 23 (23) | 13 (13) |  |
| <40 mL/min | 114 (23) | 33 (33) | 12 (12) | 21 (21) | 31 (31) | 17 (17) |  |
| <30 mL/min | 162 (32) | 23 (23) | 28 (27) | 36 (36) | 27 (27) | 48 (48) |  |
| <20 mL/min | 64 (13) | 12 (12) | 19 (19) | 15 (15) | 9 (9.0) | 9 (9.0) |  |
| <10 mL/min | 4 (0.8) | 0 | 2 (2.0) | 1 (1.0) | 1 (1.0) | 0 |  |
| Other | 2 (0.4) | 0 | 2 (2.0) | 0 | 0 | 0 |  |
| **Age threshold to define platinum ineligibility** |  |  |  |  |  |  | 0.001 |
| None | 59 (12) | 14 (14) | 17 (17) | 11 (11) | 4 (4.0) | 13 (13) |  |
| >70 yr | 48 (9.5) | 8 (7.9) | 9 (8.8) | 7 (7.0) | 18 (18) | 6 (6.0) |  |
| >75 yr | 121 (24) | 21 (21) | 17 (17) | 37 (37) | 28 (28) | 18 (18) |  |
| >80 yr | 167 (33) | 41 (41) | 32 (31) | 24 (24) | 33 (33) | 37 (37) |  |
| >85 yr | 77 (15) | 13 (13) | 18 (18) | 12 (12) | 16 (16) | 18 (18) |  |
| >90 yr | 31 (6.2) | 4 (4.0) | 9 (8.8) | 9 (9.0) | 1 (1.0) | 8 (8.0) |  |
| **ECOG PS threshold to define platinum ineligibility** |  |  |  |  |  |  | 0.059 |
| None | 4 (0.8) | 1 (1.0) | 0 | 2 (2.0) | 0 | 1 (1.0) |  |
| ECOG PS ≥0 | 3 (0.6) | 0 | 0 | 1 (1.0) | 1 (1.0) | 1 (1.0) |  |
| ECOG PS ≥1 | 40 (8.0) | 10 (9.9) | 9 (8.8) | 6 (6.0) | 10 (10) | 5 (5.0) |  |
| ECOG PS ≥2 | 204 (41) | 47 (47) | 43 (42) | 48 (48) | 37 (37) | 29 (29) |  |
| ECOG PS ≥3 | 228 (45) | 41 (41) | 40 (39) | 40 (40) | 48 (48) | 59 (59) |  |
| ECOG PS ≥4 | 24 (4.8) | 2 (2.0) | 10 (9.8) | 3 (3.0) | 4 (4.0) | 5 (5.0) |  |
| **NYHA heart failure class to define platinum ineligibility** |  |  |  |  |  |  | <0.001 |
| None | 23 (4.6) | 6 (5.9) | 3 (2.9) | 8 (8.0) | 0 | 6 (6.0) |  |
| Class I | 7 (1.4) | 1 (1.0) | 1 (1.0) | 3 (3.0) | 1 (1.0) | 1 (1.0) |  |
| Class II | 123 (24) | 31 (31) | 20 (20) | 31 (31) | 27 (27) | 14 (14) |  |
| Class III | 294 (58) | 57 (56) | 54 (53) | 50 (50) | 65 (65) | 68 (68) |  |
| Class IV | 56 (11) | 6 (5.9) | 24 (24) | 8 (8.0) | 7 (7.0) | 11 (11) |  |
| **Grade of peripheral neuropathy to define platinum ineligibility** |  |  |  |  |  |  | 0.067 |
| None | 16 (3.2) | 6 (5.9) | 1 (1.0) | 6 (6.0) | 0 | 3 (3.0) |  |
| Grade ≥1 | 16 (3.2) | 5 (5.0) | 3 (2.9) | 3 (3.0) | 2 (2.0) | 3 (3.0) |  |
| Grade ≥2 | 223 (44) | 48 (48) | 42 (41) | 49 (49) | 40 (40) | 44 (44) |  |
| Grade ≥3 | 210 (42) | 39 (39) | 42 (41) | 35 (35) | 53 (53) | 41 (41) |  |
| Grade ≥4 | 37 (7.4) | 3 (3.0) | 13 (13) | 7 (7.0) | 5 (5.0) | 9 (9.0) |  |
| Other | 1 (0.2) | 0 | 1 (1.0) | 0 | 0 | 0 |  |
| **CrCl level to determine cisplatin ineligibility** |  |  |  |  |  |  | <0.001 |
| <30 mL/min | 87 (17) | 12 (12) | 19 (19) | 13 (13) | 16 (16) | 27 (27) |  |
| <40 mL/min | 112 (22) | 25 (25) | 22 (22) | 21 (21) | 29 (29) | 15 (15) |  |
| <50 mL/min | 173 (34) | 45 (45) | 21 (21) | 38 (38) | 36 (36) | 33 (33) |  |
| <60 mL/min | 110 (22) | 14 (14) | 35 (34) | 27 (27) | 18 (18) | 16 (16) |  |
| <70 mL/min | 15 (3.0) | 4 (4.0) | 5 (4.9) | 1 (1.0) | 0 | 5 (5.0) |  |
| Other | 1 (0.2) | 0 | 0 | 0 | 0 | 1 (1.0) |  |
| I do not consider CrCl when  assessing cisplatin eligibility | 5 (1.0) | 1 (1.0) | 0 | 0 | 1 (1.0) | 3 (3.0) |  |
| 1L = first line; CrCl = creatinine clearance; ECOG PS = Eastern Cooperative Oncology Group performance status; mUC = metastatic urothelial carcinoma; NHYA = New York Heart Association. | | | | | | | |

**Supplementary Table 5.** Factors associated with reporting no threshold vs any age threshold to define platinum ineligibility

|  | **Unadjusted** | | | **Adjusted** | | |
| --- | --- | --- | --- | --- | --- | --- |
|  | **No age threshold**  **(*n =* 59)** | **Any age threshold**  **(*n =* 444)** |  | **No vs any threshold** | | |
|  | ***n* (%)** | ***n* (%)** | ***p* Value** | **Odds ratio** | **95% CI** | ***p* Value** |
| **Country** |  |  | 0.040 |  |  |  |
| Germany | 14 (14) | 87 (86) |  | — | — |  |
| Spain | 17 (17) | 85 (83) |  | 0.63 | 0.26, 1.58 | 0.3 |
| France | 11 (11) | 89 (89) |  | 0.44 | 0.16, 1.20 | 0.11 |
| Italy | 4 (4.0) | 96 (96) |  | 0.22 | 0.06, 0.73 | 0.020 |
| UK | 13 (13) | 87 (87) |  | 0.57 | 0.23, 1.43 | 0.2 |
| **Sex** |  |  | 0.058 |  |  |  |
| Male | 36 (10) | 326 (90) |  | — | — |  |
| Female | 16 (14) | 96 (86) |  | 1.37 | 0.66, 2.77 | 0.4 |
| I prefer not to answer | 7 (24) | 22 (76) |  | 4.23 | 1.36, 12.5 | 0.010 |
| **Primary specialty** |  |  | 0.9 |  |  |  |
| Oncology | 40 (12) | 307 (88) |  | — | — |  |
| Urology | 19 (12) | 137 (88) |  | 1.16 | 0.58, 2.26 | 0.7 |
| **Years in practice** |  |  | 0.014 |  |  |  |
| 3-6 | 2 (8.7) | 21 (91) |  | — | — |  |
| 7-10 | 8 (6.0) | 126 (94) |  | 0.46 | 0.10, 3.32 | 0.4 |
| 11-14 | 26 (16) | 141 (84) |  | 1.22 | 0.30, 8.21 | 0.8 |
| 15-18 | 7 (7.4) | 87 (93) |  | 0.47 | 0.10, 3.51 | 0.4 |
| >18 | 16 (19) | 69 (81) |  | 1.82 | 0.42, 12.7 | 0.5 |
| **Time spent providing direct patient care** |  |  | 0.020 |  |  |  |
| 50%-74% | 3 (3.9) | 74 (96) |  | — | — |  |
| 75%-100% | 56 (13) | 370 (87) |  | 2.11 | 0.62, 9.87 | 0.3 |
| **Practice type** |  |  | 0.15 |  |  |  |
| Private hospital | 14 (14) | 88 (86) |  | — | — |  |
| Public non-teaching hospital | 9 (7.4) | 112 (93) |  | 0.28 | 0.10, 0.73 | 0.010 |
| Public teaching hospital | 29 (14) | 174 (86) |  | 0.48 | 0.20, 1.15 | 0.097 |
| Public/private office | 3 (5.6) | 51 (94) |  | 0.14 | 0.03, 0.57 | 0.010 |
| Specialist cancer center | 4 (17) | 19 (83) |  | 0.73 | 0.16, 2.75 | 0.7 |
| **Average no. of patients with mUC treated per month** |  |  | <0.001 |  |  |  |
| ≥20 | 29 (22) | 104 (78) |  | — | — |  |
| 11-19 | 7 (5.0) | 134 (95) |  | 0.25 | 0.09, 0.62 | 0.004 |
| 2-10 | 23 (10) | 206 (90) |  | 0.36 | 0.17, 0.74 | 0.006 |
| mUC = metastatic urothelial carcinoma. | | | | | | |
